# Prevalence and risk factors associated with gastrointestinal parasites in goats (*Capra hircus*) and sheep (*Ovis aries*) from three provinces of China

**DOI:** 10.3389/fmicb.2023.1287835

**Published:** 2023-11-30

**Authors:** Weimin Cai, Cheng Cheng, Qianqian Feng, Yifei Ma, Enyu Hua, Shimin Jiang, Zhaofeng Hou, Dandan Liu, Anlong Yang, Darong Cheng, Jinjun Xu, Jianping Tao

**Affiliations:** ^1^Jiangsu Co-innovation Center for Prevention and Control of Important Animal Infectious Diseases and Zoonoses, College of Veterinary Medicine, Yangzhou University, Yangzhou, China; ^2^Rudong Animal Disease Control Center, Nantong, China; ^3^Changshu Animal Disease Control Center, Suzhou, China; ^4^Zhangjiajie Yongding District Animal Husbandry and Fishery Affairs Center, Zhangjiajie, China; ^5^Yangzhou Municipal Bureau of Agriculture and Rural Affairs, Yangzhou, China

**Keywords:** small ruminants, parasite, epidemiology, morphology, risk coefficient

## Abstract

Gastrointestinal (GI) parasites in small ruminants, especially goats and sheep, have caused significant socio-economic and public health challenges worldwide. The aim of the present study was to investigate the diversity and prevalence of GI parasites in goats and sheep in Jiangsu, Shaanxi and Hunan provinces of China, and to assess whether the age of animals, sampling season and feeding mode influence the distribution and infection of GI parasites. A total of 1,081 fecal samples collected from goats (*n* = 835) and sheep (*n* = 246) were detected by saturated saline flotation technique and nylon sifter elutriation and sieving method for eggs/oocysts, respectively. Based on the morphological observation of eggs and oocysts, one tapeworm, five nematodes, three trematodes and nineteen coccidia were identified, of which seven helminths belong to zoonotic parasites. The infection rate of parasites was 83.4% (902/1081) in total samples, 91.6% (765/835) in goats, and 55.7% (137/246) in sheep. The infection rate of coccidia was 71.0% (767/1081), and that of helminths was 56.2% (607/1081). The dominant species was *E. alijeri* (67.3%, 562/835) in goats, *E. parva* (30.1%, 74/246) in sheep. The highest prevalent helminths were Trichostrongylidae spp. in goats (58.3%, 487/835), and *Moniezia* spp. in sheep (22.76%, 56/246). Of 902 positive samples, 825 (91.5%, 825/902) contained multiple (2–10) parasites. The feeding mode, sampling season and regions were relevant risk factors which have significant influence on the occurrence of GI parasites in goats and sheep. The risk coefficient of parasite infection in autumn was 2.49 times higher than spring (Odds ratio = 2.49, 95% CI = 1.51–4.09, *p* < 0.001). Compared to raising on the high beds, the goats and sheep raising on the ground had the higher risk of parasite infection (OR = 3.91, 95% CI = 2.07–7.40, *p* < 0.001). The risk coefficient of parasite infection in Shaanxi and Hunan was 3.78 and 1.25 times higher than that in Jiangsu (OR = 3.78, 95% CI = 2.01–7.12, *p* < 0.001; OR = 1.25, 95% CI = 1.21–1.29, *p* < 0.001). These data are significant for the development of prevention strategies to minimise economic losses from small ruminant production and to reduce the risk of water and food infecting humans as vectors of zoonotic parasitic diseases.

## 1 Introduction

With rapid economic and social development, food consumption in China has undergone tremendous changes (Han et al., 2020). Mutton is more and more favored by consumers because of its advantages of low fat and cholesterol, high protein and good flavor (Van Heerden and Strydom, 2017). At the same time, ewe’s milk as a supplement to milk has also begun to follow with interest. The breeding of small ruminants, such as goats and sheep, is one of the main sources of meat production in China and plays a significant role in food security and safety, especially after the outbreak of African swine fever (Woonwong et al., 2020). Raising these animals can not only provide economic guarantee for the breeding industry, but also reduce the incidence of poverty, which is conducive to the early completion of China’s comprehensive poverty alleviation work (Briske et al., 2015).

Gastrointestinal (GI) parasitic disease is a kind of chronic consumptive disease. The growth and development of goats and sheep infected by parasites often slows down due to the predatory of nutrients by parasites or the toxic effects of parasite secretions and metabolites (Craig, 2018). Abnormal estrus, low conception rate and even abortion occurred in nanny goats and ewes and reproductive performance of bill goats and rams and survival rate of kids and lambs were decreased (Taylor et al., 2007). When the disease is serious, it is easy to cause a large number of livestock death, thus causing significant economic losses and seriously restricted the development of animal husbandry (Charlier et al., 2020). GI parasites infecting goats and sheep include helminths and protozoa, among which nematodes and coccidia are the most common (Asmare et al., 2016).

In 2020, the amount of goats and sheep on hand was 306.55 million and the production of mutton was 4.92 million tons (National Bureau of Statistics, 2021). The scale of sheep breeding continues to expand, and the feeding mode has developed from small-scale free stocking to large-scale high bed confinedness. Season has been considered as a risk factor affecting the rate of parasitic infection (Keeton and Navarre, 2018; Zajac and Garza, 2020). Several surveys have shown that GI parasites in goats and sheep are common in China, and feeding modes and seasons have an impact on the occurrence of GI parasites (Cai and Bai, 2009; Wang et al., 2010; Han et al., 2017; Yan et al., 2021), but the prevalence and risk factors of GI parasitic diseases in goats and sheep in Jiangsu, Shaanxi and Hunan provinces were not clear. The purpose of the present study was therefore to determine the prevalence, distribution and factors associated with GI parasite infection in goats and sheep from selected communities in the Jiangsu, Shaanxi and Hunan provinces of China.

## 2 Materials and methods

### 2.1 Ethical approval

All animals utilized in this research were approved by the Animal Ethics Committee of Yangzhou University.

### 2.2 Study site

**Figure 1:**
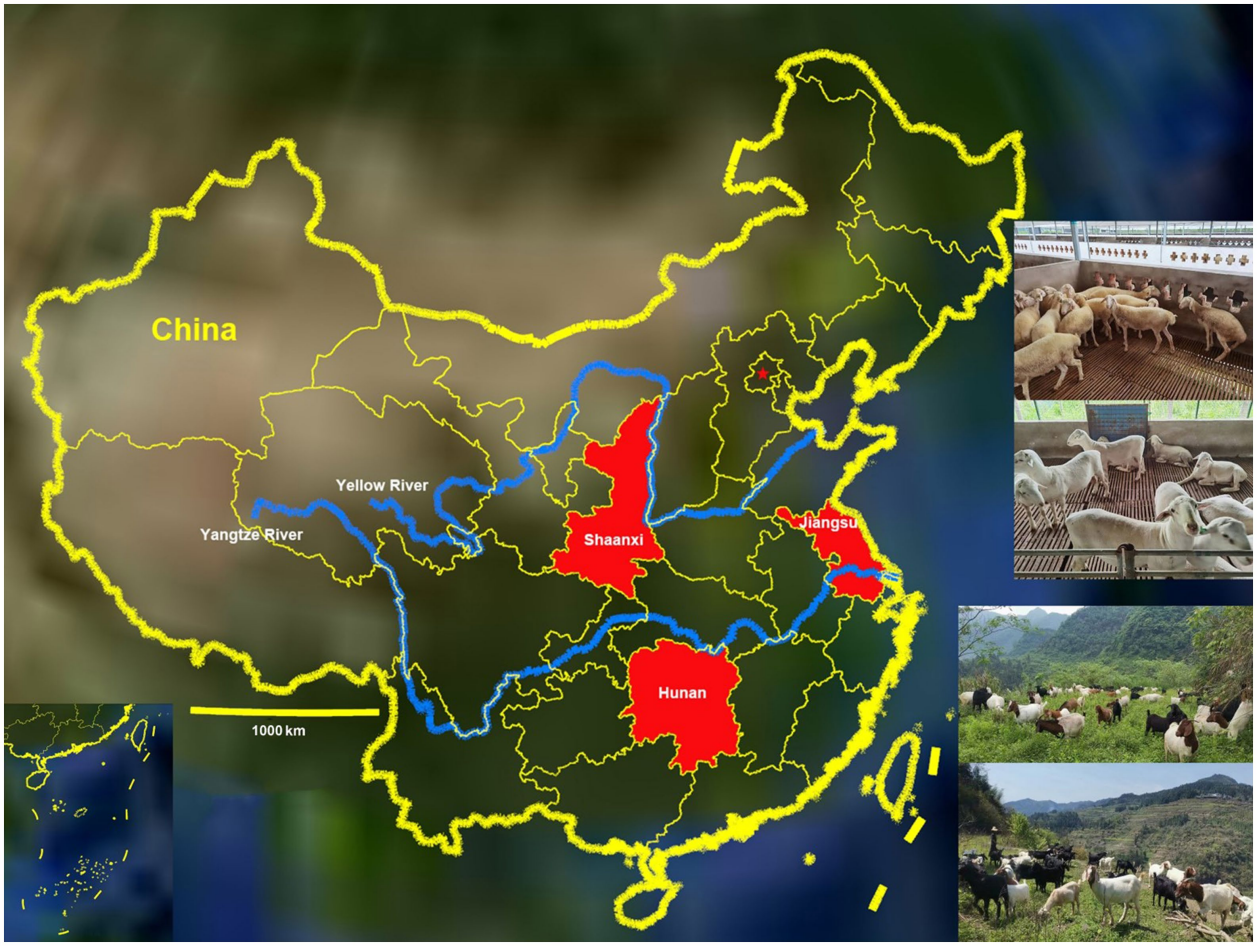
Distribution of goats and sheep farms involved in the study (red) throughout the map of China.

The study was conducted in Jiangsu, Shaanxi and Hunan, China, from September 2018 to July 2020. Jiangsu Province (116°21′ ~ 121°57′ E and 30°45′ ~ 35°08′ N) lies in the lower reaches of the Yangtze River in the east of China, Shaanxi Province (105°29′ ~ 111°15′ E and 31°42′ ~ 39°45′ N) lies in the middle reaches of the Yellow River in the northwest of China and Hunan Province (108°47′ ~ 114°15′ E and 24°38′ ~ 30°08′ N) lies in the middle reaches of the Yangtze River in the south of China (Figure 1). The average temperatures in Jiangsu, Shaanxi and Hunan are 13–16°C, 9–16°C and 16–19°C, respectively. The average annual rainfall is about 1,000 mm, 600 mm and 1,500 mm in the three provinces of Jiangsu, Shaanxi and Hunan, respectively. There are more than 3.9, 8.67 and 6.68 million goats and sheep in Jiangsu, Shaanxi and Hunan provinces, respectively.

### 2.3 Sample collection

A total of 1,081 fecal samples were collected from goats (*Capra hircus*) and sheep (*Ovis aries*) farms in Jiangsu (851), Shaanxi (180) and Hunan (50). Of them, 835 were from goats and 246 from sheep; 726 fecal samples were collected from goats and sheep raised with high bed confinedness and 355 fecal samples were collected from goats and sheep fed *ad libitum* on the ground; 433 fecal samples were collected from goats and sheep less than 6 months old, 235 from 6–12 months old, and 413 from older than 12 months old; 200 fecal samples were collected in spring, 386 in summer, 254 in autumn and 241 in winter. All feces were collected directly from rectum, about 30 g per sample. The collected samples were stored in a ~ 4°C sample box for laboratory examination.

### 2.4 Sample examination and morphological identification of parasite species

Nematode eggs, cestode eggs and coccidian oocysts were detected by the saturated saline floatation method (Taylor et al., 2007). Briefly, 10 g of goats and sheep faeces were diluted with 15 mL of saturated saline, filtered through a filter with a pore size of 250 μm, and the filtrate was centrifuged for 5 min at 800 × *g*. A coverslip was placed over the surface of the supernatant, and it was viewed under a microscope after 3 min. The eggs of trematodes were examined by nylon sieve washing method (Taylor et al., 2007), i.e., 10 g feces diluted with water passed through 60 mesh (aperture = 250 μm) sieve and 260 mesh (aperture = 57 μm) sieve successively, and then the filter residue in the 260 mesh sieve was washed with water until the final filtrate was clear. Finally, the sediment in the sieve mesh was observed under a microscope. The eggs of nematode, tapeworm and trematode were observed and identified under 40**×** objective lens. Unsporulated oocysts were incubated in 2.5% potassium dichromate (K_2_Cr_2_O_7_) for 5–7 days to form spores, the culture solution was centrifuged at 800 × *g* for 5 min, the supernatant was discarded, and the precipitate was resuspended in saturated saline and centrifuged at 700 × *g* for 4 min, and the centrifuged supernatant was aspirated for sporulation oocysts to be identified under the 100 × objective. All identifications were performed as previously described (Eckert et al., 1995; Taylor et al., 2007; Chartier and Paraud, 2012; Zajac and Conboy, 2012; Berto et al., 2014; Han et al., 2017; Macedo et al., 2019; Yan et al., 2021).

### 2.5 Statistical analysis

The potential risk factors of parasite infection rate in goats and sheep were analyzed by chi square test with IBM SPSS statistics, and the difference was judged by *p* < 0.05. All tests were 2-sided, and odds ratio (OR) and 95% confidence interval (CI) were calculated by likelihood ratio statistics, otherwise the correlation of infection rate in 3 years is not significant. Probability is the proportion of times that an outcome would occur if an experiment or observation were repeated a large number of times. A relative risk is the ratio of 2 probabilities. The odds are the probability of an event occurring, divided by the probability of that event not occurring. An odds ratio is the ratio of 2 odds.

## 3 Results

### 3.1 Species of gastrointestinal parasites

**Figure 2:**
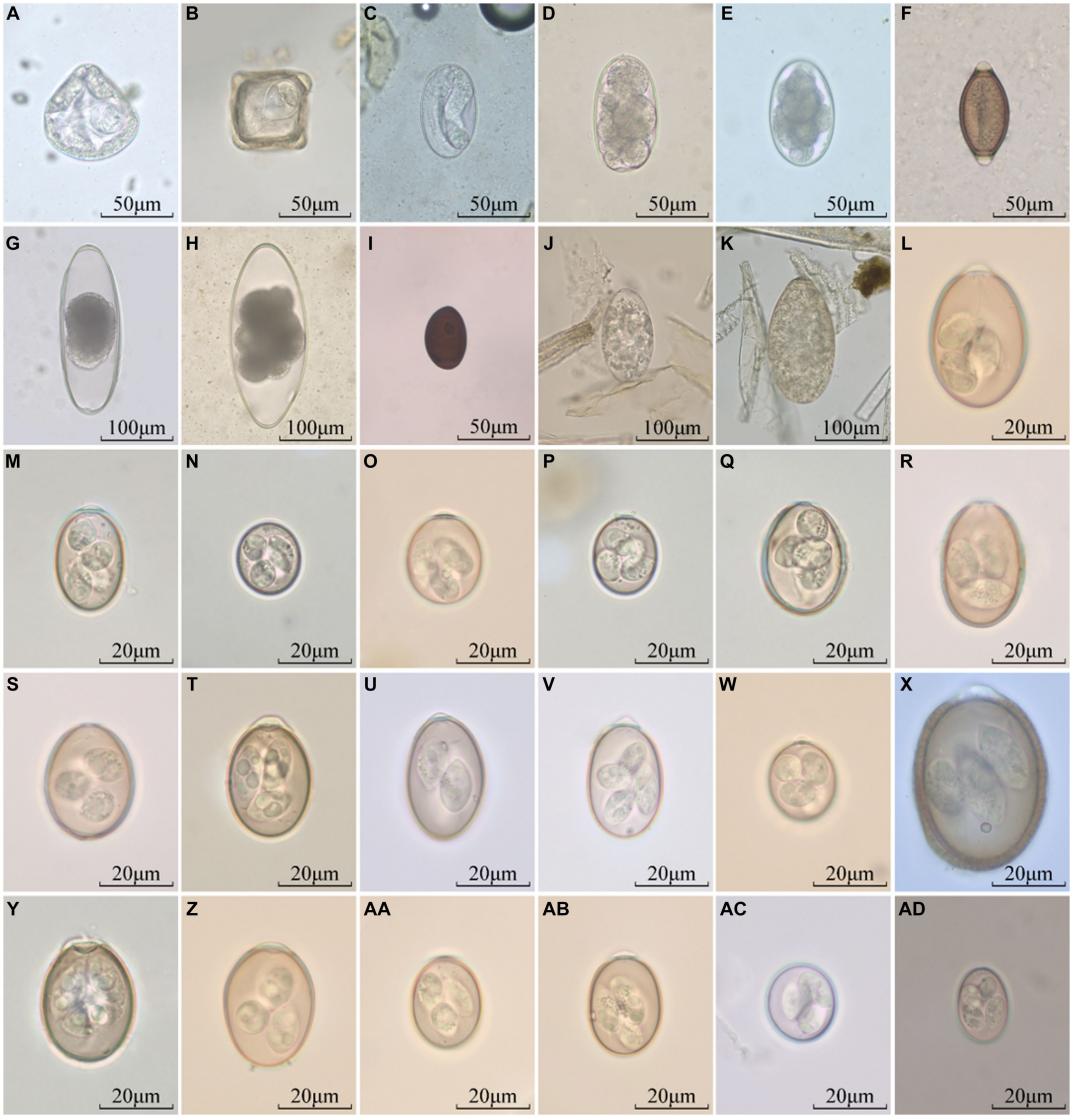
Parasites identified in fecal samples from goats and sheep. **(A,B)**: *Moniezia* spp., **(C)**: *Strongyloides* spp., **(D)**: *Bunostomum trigonocephalum*, **(E)**: Trichostrongylidae spp., **(F)**: *Trichuris* spp., **(G,H)**: *Nematodirus* spp., **(I)**: *Dicrocoelium* spp., **(J)**: *Paramphistomum* spp., **(K)**: *Fasciola* spp., **(L)**: *E. christenseni*, **(M)**: *E. arloingi*, **(N)**: *E. alijeri*, **(O)**: *E. hirci*, **(P)**: *E. ninakohly akimovae*, **(Q)**: *E. apsheronica*, **(R)**: *E. caprina*, **(S)**: *E. caprovina*, and **(T)**: *E. jolchijevi*, **(U)**: *E. ahsata*, **(V)**: *E. bakuensis*, **(W)**: *E. crandallis*, **(X)**: *E. intricate*, **(Y)**: *E. faurei*, **(Z)**: *E. granulosa*, **(AA)**: *E. ovinoidalis*, **(AB)**: *E. weybridgensis*, **(AC)**: *E. parva*, **(AD)**: *E. pallida.*

**Figure 3:**
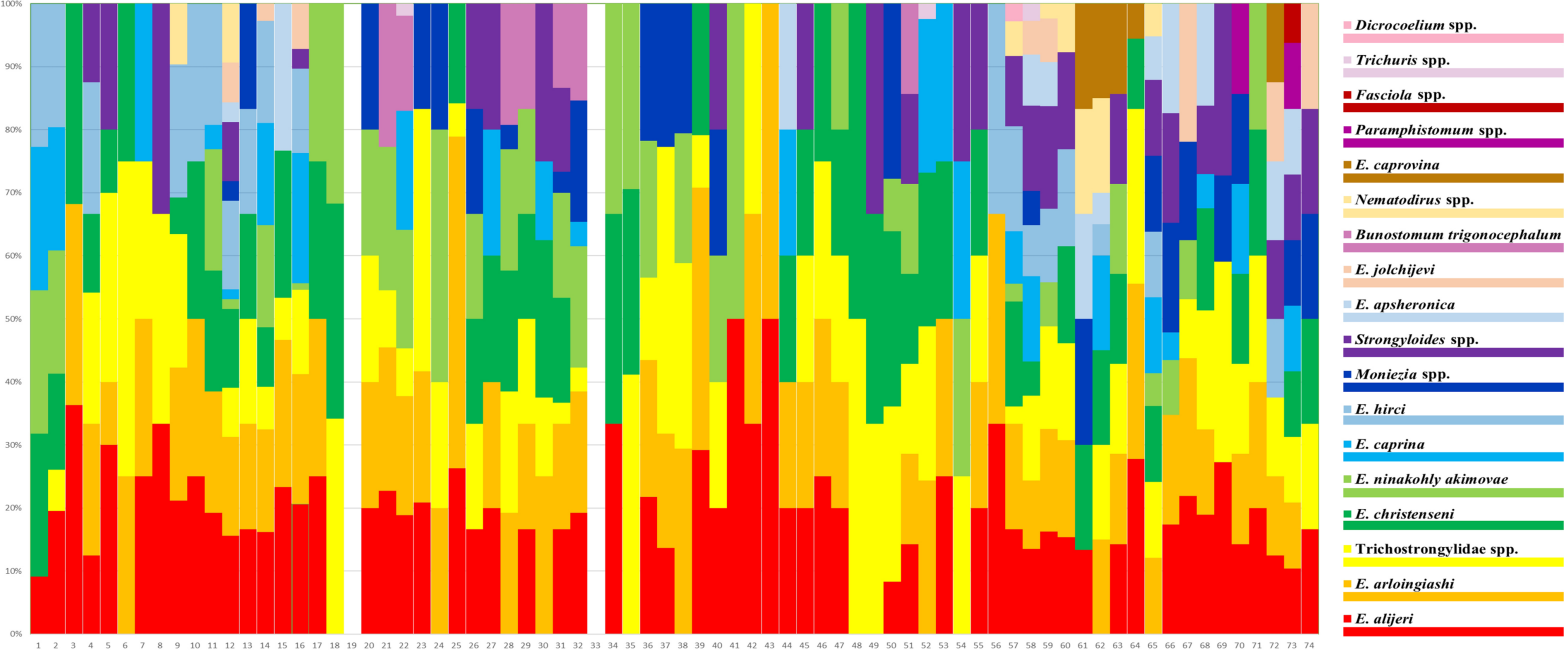
Distribution of prevalence of 18 goat GI parasites on 74 goat farms. Each bar represents one farm. The 18 parasite species are represented by 18 colours.

**Figure 4:**
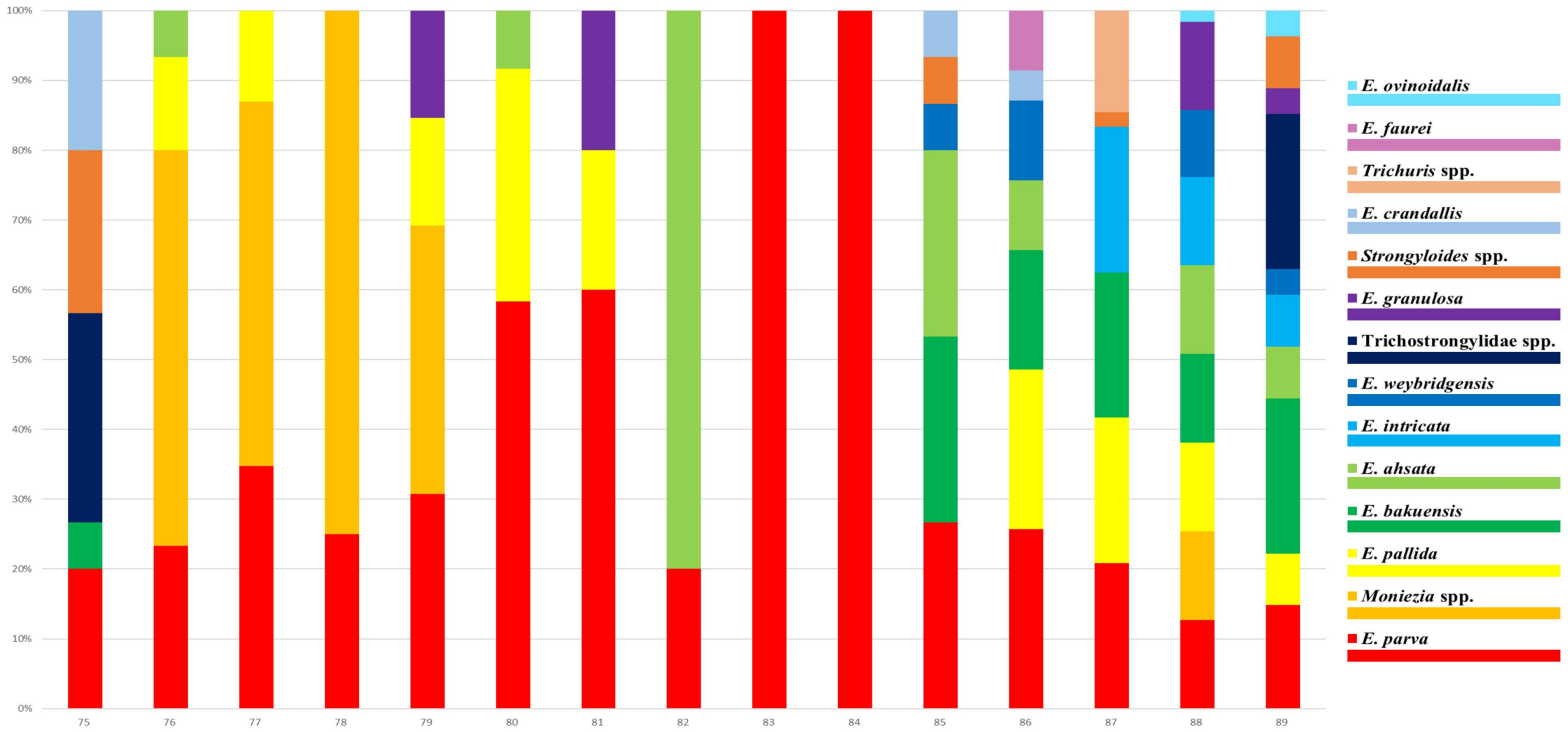
Distribution of infection rates of 14 sheep GI parasites on 15 sheep farms. Each bar represents one farm. The 14 parasite species are represented by 14 colours.

The helminth eggs and *Eimeria* oocysts observed from samples were demonstrated in Figure 2. A total of 28 species of GI parasites were observed. Eighteen species of GI parasites, including *Moniezia* spp., *Strongyloides* spp., *Bunostomum trigonocephalum*, Trichostrongylidae spp., *Trichuris* spp., *Nematodirus* spp., *Dicrocoelium* spp., *Paramphistomum* spp., *Fasciola* spp., *Eimeria christenseni*, *Eimeria arloingi*, *Eimeria alijeri*, *Eimeria hirci*, *Eimeria ninakohly akimovae*, *Eimeria apsheronica*, *Eimeria caprina*, *Eimeria caprovina* and *Eimeria jolchijevi*, were detected from goats on 74 goats farms (Figure 3). And fourteen species of GI parasites, including *Moniezia* spp., *Strongyloides* spp., Trichostrongylidae spp., *Trichuris* spp., *Eimeria ahsata*, *Eimeria bakuensis*, *Eimeria crandallis*, *Eimeria intricate*, *Eimeria faurei*, *Eimeria granulosa*, *Eimeria ovinoidalis*, *Eimeria weybridgensis*, *Eimeria parva and Eimeria pallida*, were detected from sheep on 15 sheep farms (Figure 4).

### 3.2 Prevalence and coinfection of gastrointestinal parasites

**Figure 5:**
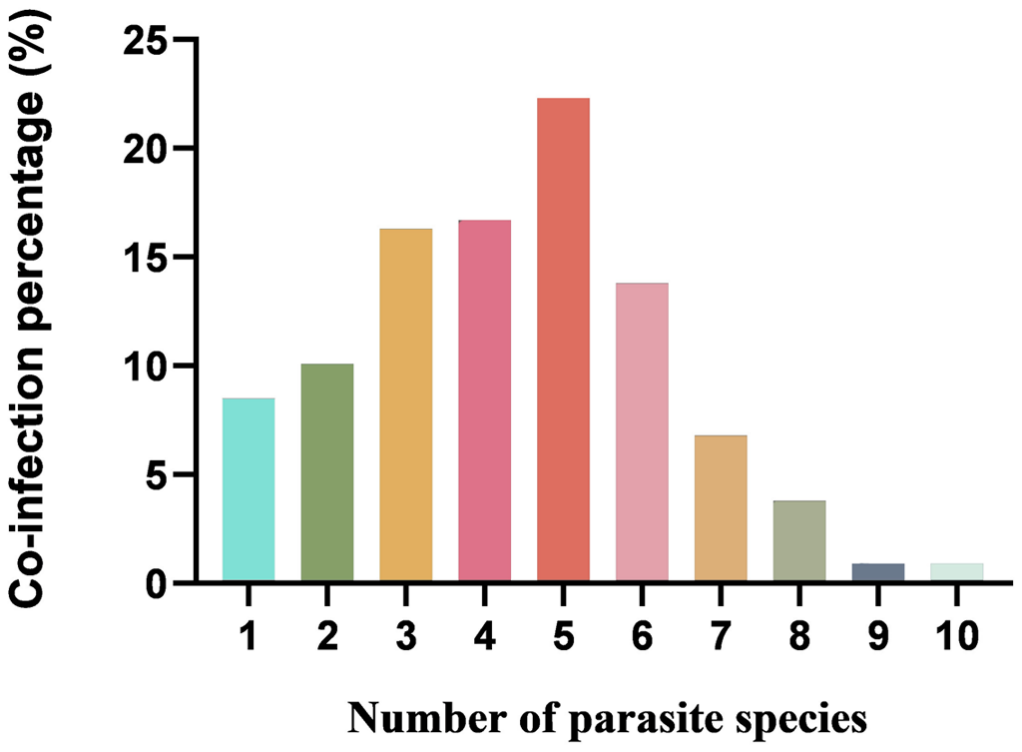
Percentage with single or mixed infections of different GI parasite species in goats and sheep in China.

Overall, 902 of 1,081 (including 835 goats and 246 sheep) fecal samples contained at least one parasite, in which the parasite infection rate was 91.6% (765/835) in goats and 55.7% (137/246) in sheep, respectively. The infection rate of helminths was 56.2% (607/1081), and the infection rate of coccidia was 71.0% (767/1081) (Table 1). Of 902 positive samples, 77 samples (8.5%, 77/902) contained one parasite, and 825 (91.5%, 825/902) contained multiple (2–10) parasites. The rates containing 2, 3, 4, 5, 6, 7, 8, 9 and 10 parasites in positive samples were 10.1% (91), 16.3% (147), 16.7% (151), 22.3% (201), 13.8% (124), 6.8% (61), 3.8% (34), 0.9% (8) and 0.9% (8), respectively (Figure 5). The dominant species in goats was *E. alijeri* (67.3%, 562/835), and the dominant species in sheep was *E. parva* (37.4%, 92/246). Regarding helminths, the infection rate of Trichostrongylidae spp. was highest (58.3%, 487/835) in goats, whereas that of *Moniezia* spp. was highest (22.76%, 56/246) in sheep (Tables 2, 3).

**Table 1:** The infection rate of helminths and coccidia in goats and sheep.

	Helminths % (n/N)	Coccidia % (n/N)	Total % (n/N)
Goats	63.5 (530/835)	78.3 (654/835)	91.6 (765/835)
Sheep	31.3 (77/246)	45.9 (113/246)	55.7 (137/246)
Total	56.2 (607/1081)	71.0 (767/1081)	83.4 (902/1081)

**Table 2:** The prevalence of gastrointestinal parasite eggs or oocysts in goats in different provinces and seasons.

Province		Jiangsu				Hunan	Shaanxi	Total
Season		Spring	Summer	Autumn	Winter	Summer	Summer	–
Sample size (N)		139	68	244	160	50	174	835
Species and prevalence (n/N)/Positive No. (n)	*Moniezia* spp.	7.2/10	0/0	16.4/40	35.0/56	60.0/30	25.3/44	21.6/180
	*Strongyloides* spp.	21.6/30	7.4/5	17.6/43	21.9/35	60.0/30	0/0	17.1/143
	*Bunostomum trigonocephalum*	7.2/10	0/0	11.5/28	15.6/25	0/0	0/0	7.5/63
	Trichostrongylidae spp.	64.7/90	26.5/18	43.9/107	56.3/90	100.0/50	75.9/132	58.3/487
	*Trichuris* spp.	0.7/1	0/0	0.4/1	0/0	0/0	0.6/1	0.4/3
	*Nematodirus* spp.	0/0	0/0	2.0/5	3.8/6	0/0	27.0/47	6.9/58
	*Dicrocoelium* spp.	0/0	0/0	0/0	0/0	0/0	0.6/1	0.1/1
	*Paramphistomum* spp.	0/0	0/0	0/0	0/0	40.0/20	0/0	2.4/20
	*Fasciola* spp.	0/0	0/0	0/0	0/0	12.0/6	0/0	0.7/6
	*E. christenseni*	64.7/90	76.5/52	46.3/113	39.4/63	80.0/40	37.4/65	50.7/423
	*E. arloingi*	53.2/74	61.8/42	69.3/169	56.3/90	80.0/40	77.6/135	65.9/550
	*E. alijeri*	55.4/77	82.4/56	61.1/149	66.9/107	100.0/50	70.7/123	67.3/562
	*E. hirci*	0/0	52.9/36	16.0/39	20.0/32	20.0/10	40.8/71	22.5/188
	*E. ninakohlyakimovae*	23.7/33	45.6/31	48.0/117	44.4/71	20.0/10	16.1/28	34.7/290
	*E. apsheronica*	7.2/10	10.3/7	0/0	1.3/2	40.0/20	38.5/67	12.7/106
	*E. caprina*	14.4/20	60.3/41	14.3/35	16.9/27	40.0/20	29.3/51	23.2/194
	*E. caprovina*	0/0	0/0	0/0	0/0	20.0/10	19.5/34	5.3/44
	*E. jolchijevi*	0/0	0/0	0/0	6.9/11	40.0/20	26.4/46	9.2/77
	Total	92.1/128	95.6/65	88.5/216	89.4/143	100.0/50	93.7/163	91.6/765

**Table 3:** The prevalence of gastrointestinal parasite eggs or oocysts in sheep in different provinces and seasons.

Province		Jiangsu				Shaanxi	Total
Season		Spring	Summer	Autumn	Winter	Summer	–
Sample size (N)		61.00	88.00	10.00	81.00	6.00	246
Species and prevalence (n/N)/Positive No. (n)	*Moniezia* spp.	8.2/5	0.00	100.0/10	50.6/41	0/0	22.76/56
	*Strongyloides* spp.	0/0	10.2/9	0/0	0/0	33.3/2	4.47/11
	Trichostrongylidae spp.	0/0	10.2/9	0/0	0/0	100.0/6	6.10/15
	*Trichuris* spp.	0/0	8.0/7	0/0	0/0	0/0	2.85/7
	*E. ahsata*	1.6/1	13.6/12	100.0/10	11.1/9	33.3/2	13.82/34
	*E. bakuensis*	0/0	18.2/16	100.0/10	14.8/12	100.0/6	17.89/44
	*E. crandallis*	0/0	8.0/7	0/0	3.7/3	0/0	4.07/10
	*E. intricata*	0/0	11.4/10	100.0/10	0/0	33.3/2	8.94/22
	*E. faurei*	0/0	0/0	0/0	7.4/6	0/0	2.44/6
	*E. granulosa*	4.9/3	0/0	100.0/10	0/0	16.7/1	5.69/14
	*E. ovinoidalis*	0/0	0/0	10.0/1	0/0	16.7/1	0.81/2
	*E. weybridgensis*	0/0	1.1/1	60.0/6	9.9/8	16.7/1	6.50/16
	*E. parva*	23.0/14	30.7/27	100.0/10	45.7/37	66.7/4	37.40/92
	*E. pallida*	11.5/7	11.4/10	100.0/10	28.4/23	33.3/2	21.14/52
	Total	36.1/22	43.2/38	80.0/8	77.8/63	100.0/6	55.69/137

**Table 4:** Risk factors of parasites infecting goats and sheep.

Sample information	Sample size	Positive No.	Negative No.	Positve rate	Odds ratio (95% CI)	*p*
Feeding mode						
High bed	726	559	167	77.06	1	
Ground	355	343	12	96.62	3.91(2.07 ~ 7.40)	<0.001
Month age						
<6	433	356	77	82.22	1	
6 ~ 12	235	194	41	82.55	1.02(0.67 ~ 1.55)	0.934
>12	413	352	61	85.23	1.24(0.86 ~ 1.79)	0.248
Season						
Spring	200	150	50	75.00%	1	
Summer	386	322	64	83.42%	1.68(1.11 ~ 2.55)	0.015
Autumn	254	224	30	88.19%	2.49(1.51 ~ 4.09)	<0.001
Winter	241	206	35	85.48%	1.96(1.21 ~ 3.17)	0.005
Province						
Jiangsu	851	683	168	80.26	1	
Shaanxi	180	169	11	93.89	3.78(2.01 ~ 7.12)	<0.001
Hunan	50	50	50	100.00	1.25(1.21 ~ 1.29)	<0.001

### 3.3 Distribution of gastrointestinal parasites amongst different provinces, age groups and feeding modes

*Moniezia* spp., *Strongyloides* spp. and Trichostrongylidae spp. in goats and sheep were more prevalent in Hunan Province (60, 60 and 100%) than in Jiangsu (17.3, 18.5 and 49.9%) and Shaanxi (24.4, 1.1 and 76.7%) Provinces, respectively. The occurrence of *Trichuris* spp. was higher in Jiangsu Province (1.1%, 9/851) than in Shaanxi Province (0.6%, 1/180). *B. trigonocephalum* (5.8%, 63/1081) was only found in Jiangsu Province, *Dicrocoelium* spp. (0.1%, 1/1081) was only found in Shaanxi Province, and *Paramphistomum* spp. (1.9%, 20/1081) and *Fasciola* spp. (0.6%, 6/1081) were only found in Hunan Province, respectively. Gastrointestinal parasites were more prevalent in adults >12 months (85.23%, 352/413) than in 6–12 months (82.55%, 194/235) and young animals <6 months (82.22%, 356/433), respectively.

*Dicrocoelium* spp., *Paramphistomum* spp. and *Fasciola* spp. were only found in livestock from the ground feeding mode, the rest of the identified parasites were occurred in livestock both from the high bed and ground feeding mode. Gastrointestinal parasites were more prevalent in ground feeding mode (96.62%, 343/355) than in high bed feeding mode (77.06%, 559/726).

### 3.4 Analyses of risk factors of parasites infection in goats and sheep

The effects of four risk factors on GI parasites were analyzed, including the feeding modes and month ages of goats and sheep, seasons, and regions. The risk coefficient of parasite infection in goats and sheep raised on ground was 3.91 times higher than those raised on high bed, and the difference was significant (OR = 3.91, 95% CI = 2.07–7.40, *p* < 0.001). The risk coefficient of parasite infection in 6–12 months old and > 12 months old goats and sheep was 1.02 and 1.24 times higher than that in <6 months old goats and sheep, but the difference was not significant (OR = 1.02, 95% CI = 0.67–1.55, *p* = 0.934; OR = 1.24, 95% CI = 0.86–1.79, *p* = 0.248). The risk coefficient of parasite infection in goats and sheep in summer, autumn and winter was 1.68, 2.49 and 1.96 times higher than that in spring, and the difference was significant (OR = 1.68, 95% CI = 1.11–2.55, *p* = 0.015; OR = 2.49, 95% CI = 1.51–4.09, *p* < 0.001; OR = 1.96, 95% CI = 1.21–3.17, *p* = 0.005). The risk coefficient of parasite infection in goats and sheep in Shaanxi and Hunan was 3.78 and 1.25 times higher than that in Jiangsu, and the difference was significant (OR = 3.78, 95% CI = 2.01–7.12, *p* < 0.001; OR = 1.25, 95% CI = 1.21–1.29, *p* < 0.001).

## 4 Discussion

A total of 28 GIl parasites were identified in this study, of which seven have been reported to infect humans, including *Haemonchus contortus* (Ghadirian and Arfaa, 1973; Bethony et al., 2006), *Trichostronglus* spp. (Yong et al., 2007), *Trichuris* spp. (Else et al., 2020; Polakovicova et al., 2023), *Strongyloides* spp. (Nutman, 2017), *Dicrocoelium* spp. (Lavilla-Salgado et al., 2021; Chai et al., 2009; Zeng et al., 2010; Sah et al., 2017; Lavilla-Salgado et al., 2021; Mas-Coma et al., 2022; Zarei et al., 2022), *Paramphistomum* spp. (Chai et al., 2009; Zeng et al., 2010) and *Fasciola* spp. (Sah et al., 2017; Mas-Coma et al., 2022), respectively. These pathogens can cause zoonotic diseases and pose a major threat to human public health and food safety. Patients infected with these diseases usually present with symptoms such as stomach pain, bloating and occasionally diarrhoea (Yong et al., 2007). In addition, the levels of eosinophils in blood are usually increased due to infection of these worms.

The overall prevalence of GI parasites in goats and sheep was 83.4% (902/1081), and 91.6% (765/835) goat fecal samples and 55.7% (137/246) sheep fecal samples contained at least one parasite, respectively. Similar infection rate was observed in small ruminants in the previous researches (Koinari et al., 2013; Han et al., 2017; Mohammedsalih et al., 2019; Squire et al., 2019; Paul et al., 2020; Dahourou et al., 2021). In the present study, infection rate was higher than that in Columbia (Leon et al., 2019) and Turkey (Akyuz et al., 2019), and lower than that in Britain (Pacheco et al., 2021), Ghana (Owusu et al., 2016) and Ningxia and Xinjiang of China (Cai and Bai, 2009; Yan et al., 2021). The reasons for these differences may be related to risk factors, including feeding environment, sampling season. In addition, our results revealed that 91.5% (825/902) samples contained multiple parasites, in which the number of samples mixed infection with five species of parasites was the highest (22.3%, 201/902). Mysteriously, the samples mixed infection with one species of helminths and four coccidian species were the most prevalent (55.22%, 111/201), which was consistent with a previous report (Squire et al., 2019). Co-infections with pathogens within the same host are common, and there is increasing evidence that co-infections with these pathogens may alter susceptibility to other important pathogens, such as bacteria and viruses (Viney and Graham, 2013; Mabbott, 2018).

There are many species of nematodes parasitized in the digestive tract of goats and sheep, which often present as mixed infections (Zajac and Garza, 2020). Because the eggs of *Haemonchus contortus*, *Ostertagia* spp., *Trichostronglus* spp. (belonging to Trichostrongylidae), and *Oesophagostomum* spp. (belonging to Chabertiidae) possess similar size (73 ~ 95 μm × 34 ~ 50 μm), morphology (ovoid in shape) and structure (containing numbers of embryo cells), it is difficult to distinguish between these species (Ministry of Agriculture, Fisheries and Food, 1977). In the present study, therefore, the eggs with the above morphological structure were uniformly classified as Trichostrongylidae spp. *Nematodirus* spp. parasitizes in the small intestine of ruminants such as cattle, goats and sheep, and their eggs are large, ovoid and colourless and twice the size of the typical trichostrongyle egg, and are easy to distinguish from other nematode eggs. Here, 30 eggs of *Nematodirus* spp. were measured from the samples of Shaanxi and Jiangsu, respectively. The result showed that the average size of the eggs was 250.07 μm × 111.15 μm in Shaanxi and 274.19 μm × 108.83 μm in Jiangsu, both of which was longer than that (200 μm long) reported in Inner Mongolia of China (Wang, 2018). The differences in egg size may imply that the species of *Nematodirus* spp. is different in these three areas.

There are two species of *Bunostomum* spp., including *B. trigonocephalum* in goats and sheep, and *B. phlebotomum* in cattle. The eggs of *Bunostomum* spp. are irregular broad elipse in shape, with dissimilar sidewalls and 4–8 blastomeres. The common species of *Trichuris* spp. parasitized in goats and sheep include *Trichuris ovis* and *T. globulosa*. The characteristic eggs are lemon shaped with a conspicuous plug at both ends; in the faeces these eggs appear yellow or brown in colour. Therefore, *Bunostomum* spp. and *Trichuris* spp. eggs are easy to identify under the microscope. In the present study, both *B. trigonocephalum* and *Trichuris* spp. eggs were detected, but both had low prevalence, namely the infection rate of *B. trigonocephalum* was 7.5% and zero in goats, and those of *Trichuris* spp. was 0.4 and 2.85% in sheep. Our results were consistent with the previous reports in Qinghai and Xinjiang of China (Wang et al., 2021; Yan et al., 2021). In addition, *Strongyloides* spp. eggs are oval, thinshelled and small, being half the size of typical strongyle eggs. In herbivores it is the larvated egg which is passed out in the faeces. Thus, *Strongyloides* spp. eggs are also easy to identify under the microscope. In this study, the infection rate of *Strongyloides* spp. eggs was 17.1% (143/835) in goats, and 4.47% (11/246) in sheep respectively, which was lower than that (28%) in fat-tailed sheep in Bangladesh reported by Islam et al. (2019).

*M. expansa* and *M. benedeni* are the common species of the genus *Moniezia*. It is generally agreed that *M. expansa* parasitizes in goats and sheep, occasionally cattle, and *M. benedeni* parasitizes in cattle; the eggs of *M. expansa* present irregularly triangular, whereas the eggs of *M. benedeni* appear as irregularly quadrangular; the eggs of *M. benedeni* are slightly larger than those of *M. expansa*. In the present study, both of the irregularly quadrangular and irregularly triangular eggs were observed in goats and sheep, and were uniformly identified as *Moniezia* spp. The infection rate of *Moniezia* eggs were 21.6% (180/835) in goats and 22.76% (56/246) in sheep, higher than that in Portugal (3.1%) and India (5.8%) (Bansal et al., 2015; Ruano et al., 2019).

According to data,^1^ trematodes parasitized in goats and sheep contain 13 genera in Jiangsu, and 16 genera in Shaanxi and Hunan, respectively. However, in this study, only three species of trematodes including *Dicrocoelium* spp., *Paramphistomum* spp. and *Fasciola* spp. are found in goats, and their infection rates were 0.1% (1/835), 2.4% (20/835) and 0.7% (6/835) respectively, which were lower than that in Egypt and Ghana (Amer et al., 2016; Squire et al., 2019). A possible reason for the low prevalence of trematodes in recent years is that changes in management and feeding methods reduce ingestion of infectious stages by goats and sheep. In addition, among three trematodes detected in this study, *Dicrocoelium* spp. was only found in the Shaanxi region, whereas *Paramphistomum* spp. and *Fasciola* spp. were only found in the Hunan region. *Dicrocoelium* spp. needs a first intermediate host (the land snails, shuch as Fruticicolidae) and a second intermediate host (ants, such as *Pheidole*) to complete its life cycle, and its acercariae shed from the land snails develop to infective metacercariae in ants, whereas *Paramphistomum* spp. and *Fasciola* spp. use the amphibious snails (such as *Lymnaea*) as an intermediate host, and their acercariae shed from the snails form the infective metacercariae on grass blades. Shaanxi Province spans the northwest and southwest of China, and its terrain is composed of various landforms such as plateaus, mountains, plains, and basins. The Loess Plateau accounts for 40% of the whole land area of Shaanxi Province, which are be conducive to the distribution of the land snails and ants. Therefore, the grazing goats and sheep are most likely to eat ants, resulting in *Dicrocoelium* spp. infection (Al Amin and Wadhwa, 2023). Hunan has a humid continental and subtropical monsoon climate and are surrounded by mountains and hills in the east, west and south, and there are many lowlands and swamps among mountains and hills, which are favorable for the distribution of the amphibious snails and facilitates the transmission of *Paramphistomum* spp. and *Fasciola* spp. (Taylor et al., 2007).

Intestinal coccidiosis, caused by parasites of the genus *Eimeria*, is one of the most important parasitic diseases of small ruminants worldwide (Alcala-Canto et al., 2020). To date, 17 and 15 *Eimeria* species have been described in goats and sheep, respectively (Levine, 1985; Macedo et al., 2019). In China, nine and 12 *Eimeria* species have been found in goats and sheep, respectively (Wang et al., 2010; Chartier and Paraud, 2012). Currently, nine coccidian species were recognized in goats and 10 in sheep. The infection rate was 78.3% (654/835) in goats and 45.9%(113/246) in sheep, and the overall infection rate was 71.0% (767/1081), which were consistent with the previous studies in UK, Egypt, Brazil and Algeria (Macedo et al., 2019; El-Alfy et al., 2020; Al-Neama et al., 2021), and was higher than that reported in southern Spain and northeastern China (Wang et al., 2010; Carrau et al., 2018). The dominant coccidia species was *E. arloingi* in goats, and *E. parva* in sheep respectively, which were consistent with the previous reports in the USA (Penzhorn et al., 1994; Zhao et al., 2012), Turkey (Cai and Bai, 2009; Akyuz et al., 2019), Shaanxi and Ningxia of China (Penzhorn et al., 1994; Zhao et al., 2012); (Cai and Bai, 2009; Macedo et al., 2019). *E. arloingi*, *E. ninakohlyakimovae* and *E. christenseni* in goats and *E. ovinoidalis*, *E. ahsata* and *E. bakuensis* in sheep are the most pathogenic (Bangoura and Bardsley, 2020), which also were detected in the present study. However, only one kid with mixed infection of multiple nematodes and coccidia developed diarrhea symptoms, all the other animals had no clinical symptoms of diarrhea. These coccidia can cause parasitic infections in other goats and sheep by contaminating water sources, while the infective cysts of trematodes attach to water plants and can be ingested by goats and sheep as food, triggering parasite transmission. In addition, goats and sheep infected with zoonotic trematodes and nematodes may be ingested by humans when processed as agricultural products, posing a public health threat.

Our results revealed that the risk of GI parasite infection in goats and sheep raised on ground was higher than those raised on high bed (*p* < 0.001); the infection rate of GI parasites in autumn was significantly higher than that in spring (*p* < 0.001); the risk of GI parasite infection in Shaanxi and Hunan region were lower than that in Jiangsu (*p* < 0.001), and the risk of goats and sheep being infected with GI parasites in Shaanxi region was almost four times higher than in Jiangsu region. Our results were consistent with the previous studies (Haile et al., 2010; Khalafalla et al., 2011; Han et al., 2017). The possible reasons for these results are that the number of infective eggs (or larvae) and coccidian oocysts in the pens is reduced as feces are easily removed from the slatted floor. The infective larvae (L3) of nematodes are difficult to survive in processed feeds (Torres-Acosta and Hoste, 2008). Additionally, the mild and humid environment in summer and autumn are beneficial to the development of eggs or larvae of helminths and coccidian oocysts, whereas the number of infectious eggs or larvae may decrease greatly after crossing the cold winter (Wang et al., 2020). Interestingly, the risk of sheep infected with parasites in Shaanxi Province with low temperatures and low rainfall is higher than that in Jiangsu Province, which may indicate that compared to temperature and humidity, a higher correlation presents between parasite infection in goats and sheep and breeding patterns. Moreover, the number of goats and sheep in Shaanxi (8.67 million) is greater than that in Jiangsu (3.9 million), indicating that high feeding density is more prone to the spread of parasitic diseases.

In the present study, we aimed to investigate and clarify the prevalence and risk factors of GI parasites in goats and sheep in Jiangsu, Shaanxi and Hunan provinces. Unfortunately, some intestinal protozoa such as *Cryptosporidium* spp. and *Enterocytozoon* spp., which is difficult to identify using a microscope, were not detected in this study. In addition, the species of helminths detected in this study had not been identified use molecular methods. However, the data herein presented are important for understanding the factors that influence the occurrence of the infection by these GI parasites and for the development of prophylactic strategies suitable for the different conditions. For example, the breeding pattern of goats and sheep could be changed from raising on the ground to raising on the high beds, and the goats and sheep should be routinely treated with anthelmintic drugs and/or anticoccidial drugs, in which the trematode and cestode infection can be treated using praziquantel, the nematode infection using ivermectin or albendazole, and the coccidian infection using monensin (Taylor et al., 2007; Nutman, 2017; Mas-Coma et al., 2022). These methods would minimize the economic losses for small ruminant production and reduce the risk of zoonotic parasite infection in humans.

## 5 Conclusion

This study was the first to investigate the diversity and prevalence of GI parasites in goats and sheep in Jiangsu, Shaanxi and Hunan provinces of China, and to revealed that feeding mode, sampling season and regions were relevant risk factors which have significant influence on the occurrence of GI parasites in goats and sheep. These data are of great significance for developing prevention strategies to minimize economic losses in small ruminant production and to prevent zoonotic parasite infection in humans of Jiangsu, Shaanxi and Hunan regions.

## Data availability statement

The raw data supporting the conclusions of this article will be made available by the authors, without undue reservation.

## Ethics statement

The animal studies were approved by Animal Ethics Committee of Yangzhou University. The studies were conducted in accordance with the local legislation and institutional requirements. Written informed consent was obtained from the owners for the participation of their animals in this study.

## Author contributions

WC: Data curation, Investigation, Methodology, Validation, Writing – original draft, Writing – review & editing. CC: Writing – review & editing, Investigation. QF: Investigation, Writing – review & editing. YM: Writing – review & editing. EH: Investigation, Writing – review & editing. SJ: Investigation, Writing – review & editing. ZH: Investigation, Writing – review & editing. DL: Data curation, Writing – review & editing. AY: Data curation, Writing – review & editing. DC: Data curation, Funding acquisition, Writing – review & editing. JX: Data curation, Writing – review & editing. JT: Conceptualization, Funding acquisition, Project administration, Writing – review & editing.

## Footnotes

^1^ https://portal.tdrc.org.cn/matmainView
